# A Certificateless Encryption-Based Authentication Protocol for Low Earth Orbit Satellite Network

**DOI:** 10.3390/s26154958

**Published:** 2026-08-05

**Authors:** Zhengwei Lv, Zhengxin Tao, Qingpeng Yang, Ziqi Zheng, Jiaxi Zhou, Lixia Xiao

**Affiliations:** School of Cyber Science and Engineering, Huazhong University of Science and Technology, Wuhan 430074, China; m202472269@hust.edu.cn (Z.L.); m202572335@hust.edu.cn (Z.T.); yangqingpeng@hust.edu.cn (Q.Y.); zhengziqi@hust.edu.cn (Z.Z.); zhoujiaxi@hust.edu.cn (J.Z.)

**Keywords:** low earth orbit satellite network, access authentication, group handover

## Abstract

In this letter, a novel access and handover authentication scheme is proposed for Low Earth Orbit (LEO) satellite networks, aiming to achieve lightweight computation, privacy preservation, and seamless secure handover for massive sensor users. Different from traditional certificate-based and pairing-dependent authentication mechanisms, the proposed scheme is constructed on certificateless cryptography, which eliminates expensive bilinear pairing operations and avoids the complex certificate management overhead in traditional public-key systems. Meanwhile, it adopts lightweight elliptic curve point addition to further lower computational burden and adapt to the limited on-board computing resources of LEO satellites. To tackle the severe signaling storm and frequent handover dilemma caused by massive, densely deployed sensor terminals, a location-aware, user-grouping-based collaborative handover authentication protocol is specifically designed. The proposed scheme rigorously satisfies essential security properties, including mutual authentication, anonymous identity privacy, known-key security, and master key forward secrecy. Simulation results verify that the proposed protocol achieves superior security performance and remarkably reduces signaling interaction overhead. In the evaluated simulation setting, the proposed mechanism achieves up to 69.81% improvement in handover success rate compared with the standalone handover scheme under high-density access conditions.

## 1. Introduction

The Low Earth Orbit (LEO) satellite network has evolved into a pivotal cornerstone of next-generation global information and communication infrastructure. By enabling deep integration and collaborative networking between space satellite constellations and terrestrial communication systems, LEO satellite networks build a ubiquitous high-speed transmission framework that breaks the coverage limitation of traditional ground networks [1]. Such space–ground integrated networking supports anytime, anywhere on-demand access for global terminals, delivering wide-coverage, stable, and high-efficiency communication services for remote and underserved areas [2]. Furthermore, LEO satellite constellations perfectly match the core performance indicators of sixth-generation (6G) networks, including ultra-low latency, broadband transmission, and ultra-high reliability. Particularly, LEO satellite networks can provide reliable long-range communication access for large-scale distributed sensor networks and massive low-power sensor nodes widely deployed in wilderness, unattended industrial zones, and ecological monitoring regions. Accordingly, LEO satellite networks have emerged as a key enabling technology for realizing full-dimensional global coverage in future 6G communications [3].

With the rapid scale expansion of LEO satellite constellations and the explosive growth of Internet of Things (IoT) sensor terminals, secure and lightweight access authentication has become a bottleneck restricting network reliable operation. The inherent broadcast openness, time-varying topology, and high orbital mobility of LEO satellites make wireless transmission links more vulnerable to eavesdropping, message forgery, replay attacks, and privacy leakage. Therefore, researching high-security, low-overhead, and scalable access and handover authentication mechanisms is not only crucial for guaranteeing normal network operation but also a necessary foundation for the sustainable construction of space–ground integrated information infrastructure. In this context, designing dedicated authentication protocols tailored to the dynamic characteristics and resource constraints of LEO satellite networks is of significant theoretical and practical value [4,5].

Distinct from static terrestrial networks, LEO satellite networks feature wide-area coverage, high-speed satellite movement, and heterogeneous space–ground hybrid architecture. These inherent characteristics make conventional terrestrial authentication schemes difficult to deploy and apply directly. LEO communication scenarios face unique challenges, such as propagation latency, intermittent link interruption, and frequent inter-satellite handovers induced by high-velocity orbital motion [6]. Especially with the massive access of dense sensor users, a large number of adjacent terminals simultaneously initiate handover requests, which easily trigger severe signaling storms, aggravate network congestion, degrade authentication efficiency, and seriously affect overall communication quality. Hence, it is urgent to develop a scalable group-aware handover authentication mechanism to accommodate massive concurrent access in LEO satellite networks [7].

In recent years, a variety of access authentication protocols have been extended and developed for satellite communication scenarios. For example, the scheme in [8] adopts symmetric cryptography to realize efficient user authentication and temporary identity update, yet it suffers from poor scalability compared with public-key authentication frameworks. The lightweight authentication protocol in [9] is optimized for satellite scenarios but still cannot resist denial-of-service and replay attacks effectively. Although the improved design in [10] enhances authentication efficiency, it remains vulnerable to offline password guessing and replay threats [11]. The work in [12] introduces clock synchronization and mutual authentication to mitigate partial security risks. In addition, ref. [13] aggregates neighboring users into groups to optimize handover signaling interaction, which relieves the signaling storm to a certain extent.

Nevertheless, most existing studies separate access authentication and handover authentication design, ignoring the joint optimization requirement under massive sensor user access. Moreover, the majority of conventional schemes either rely on heavy bilinear pairings or introduce complicated certificate management overhead, which cannot well adapt to the limited on-board computation and storage resources of LEO satellites. Meanwhile, they lack targeted grouping optimization for dense concurrent handover scenarios, making it difficult to simultaneously satisfy the triple requirements of high security, low overhead, and seamless handover in large-scale LEO satellite IoT scenarios.

Against the background, the contributions of this letter are summarized as follows:

- We propose a novel certificateless lightweight authentication framework that eliminates computationally expensive bilinear pairing operations and avoids the complicated distribution and maintenance of public key certificates. We further integrate a dynamic temporary key update mechanism to generate a unique independent session key for each communication session, which effectively prevents key leakage and improves forward secrecy as well as terminal identity privacy. Nevertheless, compared with traditional symmetric-key lightweight schemes, the proposed certificateless mechanism still introduces additional elliptic-curve point multiplication overhead to achieve stronger security properties and certificate-free trust management, reflecting a practical tradeoff between computational efficiency and security robustness in LEO satellite network environments.
- We design a location-aware user grouping and secret sharing mechanism tailored for massive sensor terminals. By clustering geographically adjacent users into groups to implement collaborative group handover authentication, we significantly reduce redundant interactive signaling, mitigate the signaling storm triggered by massive concurrent handovers, and substantially improve handover efficiency under dense user deployment scenarios. However, the effectiveness of the proposed group handover optimization mainly relies on large-scale dense-user mobility scenarios with spatial correlation. In sparse-access or low-mobility environments where user grouping advantages become less significant, the performance gain of the proposed mechanism may be reduced, and the protocol mainly retains its lightweight certificateless authentication characteristics.
- We conduct comprehensive numerical simulations to evaluate the performance in terms of security, computational overhead, signaling cost, and handover success rate. The simulation results verify that our scheme achieves superior overall performance compared with existing mainstream protocols, and it is well-suited for secure access and seamless handover of massive IoT terminals in LEO satellite networks.

## 2. System Model

In this section, we constructed an access authentication system model and an attacker model, designed the access authentication process, and analyzed its security requirements.

### 2.1. Access Authentication System Architecture

**Figure 1:**
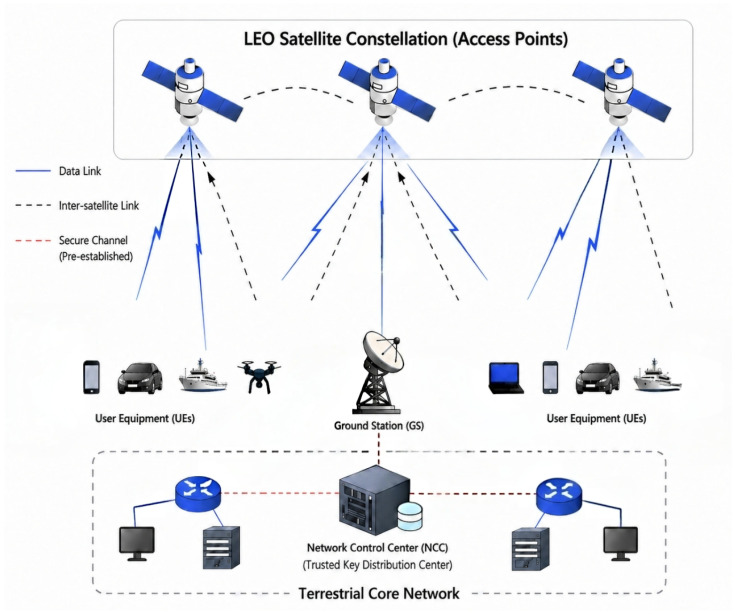
System architecture diagram.

The overall architecture of the system designed in this chapter is suitable for the secure and efficient access authentication scheme for the satellite–ground network, as shown in Figure 1.

The proposed system architecture consists of multiple core and interdependent network entities, mainly including User Equipment (UE), Access Point (AP), Ground Station (GS), and Network Control Center (NCC). Each entity undertakes independent functional tasks while cooperating closely to guarantee secure access, reliable data transmission, and orderly network operation within the LEO satellite network. Among them, the NCC acts as the core central control node of the entire LEO satellite network. It is primarily in charge of unified management, dynamic maintenance, and real-time update of global network configuration parameters. Meanwhile, the NCC also functions as a trusted Key Distribution Center (KDC). All distributed network entities must complete legitimate identity registration with the NCC in advance, so as to acquire standardized cryptographic materials and essential security parameters required for subsequent identity authentication, key negotiation, and secure communication.

The AP in this framework is deployed based on LEO satellite constellations, which undertake the core task of receiving and processing multi-terminal access requests from ground user equipment. Benefiting from the continuous improvement of on-board computing and storage capabilities of modern satellites, the LEO satellite-based AP is no longer limited to simple signal forwarding. Instead, it is capable of executing complex cryptographic operations, completing efficient identity verification and access permission judgment for massive access users, and thus effectively improving the overall response efficiency of satellite access authentication.

As a vital transmission bridge connecting space satellites and terrestrial communication systems, the GS serves as a professional communication gateway. It mainly undertakes key tasks such as signal transceiving, data forwarding, protocol conversion, and information interaction between satellite constellations and ground backbone networks, ensuring stable and high-speed data interaction across space and ground. Finally, UE refers to various distributed user terminals and sensor devices that need to access LEO satellite network. These terminals are widely distributed in complex remote scenarios and rely on satellite access services to achieve long-distance network connections and data interaction.

In this protocol model, UE interacts solely with the AP for network access, so we assume that secure communication channels have already been established between the NCC, GS, and AP.

### 2.2. Adversary Model

Given the inherent openness and broadcast nature of satellite-to-ground communication channels, the security of data transmission and user access in LEO satellite internet faces significant challenges. Unlike closed terrestrial networks, satellite communication signals propagate through open space without physical isolation, making them vulnerable to malicious exploitation. Malicious adversaries with certain technical capabilities can easily exploit this vulnerability to launch various attacks, endangering the confidentiality, integrity, and availability of network services. Specifically, adversaries may intercept and eavesdrop on confidential messages between users and satellites, stealing sensitive data such as user identities and authentication credentials; they may also fabricate fake access requests, replay valid historical messages, or tamper with data transmissions, thereby bypassing access authentication, gaining unauthorized network access, and disrupting normal communication services.

To formally characterize adversarial capabilities and attack behaviors, and conduct rigorous security analysis of the proposed authentication protocol, this paper adopts the widely used Dolev–Yao (DY) threat model [14]. As a mature theoretical framework for cryptographic protocol security analysis, the DY model is widely applied in satellite communication, wireless sensor networks, and other scenarios. It comprehensively models typical adversarial behaviors in open communication environments, including message interception, message modification, message injection, and message replay. Additionally, the model assumes the adversary has full control over the communication channel, enabling manipulation of message transmission timing and routes—consistent with the actual security risks of open satellite-to-ground communication. Adopting this model allows accurate evaluation of the proposed protocol’s ability to resist various malicious attacks.

### 2.3. User Grouping Model

To support scalable collaborative handover authentication in dense LEO satellite scenarios, a location-aware user grouping mechanism is introduced. Neighboring users with geographical proximity are dynamically clustered into the same handover group. Specifically, each group is associated with a Group Header (GH), which is responsible for collecting handover requests, aggregating secret shares, and coordinating group authentication procedures. To ensure manageable signaling coordination and stable group structure, the geographical coverage of each group is spatially constrained. For a given group $G_{k}$, all member users must satisfy the following geographical constraint: $G_{k}=UE_{i}|d\left(UE_{i},GH_{k}\right)\leq R_{g}$ where d(·) denotes the Euclidean geographical distance between the user and the GH, and $R_{g}$ represents the maximum effective group coverage radius. Considering the limited beam coverage and high mobility characteristics of LEO satellites, the group radius is further constrained by the satellite beam footprint: $R_{g}\leq \eta R_{beam},\;0<\eta <1$ where $R_{beam}$ denotes the coverage radius of the serving satellite beam, and η is the spatial safety factor used to prevent excessive group expansion near beam boundaries.

## 3. Proposed Authentication Protocol

**Figure 2:**
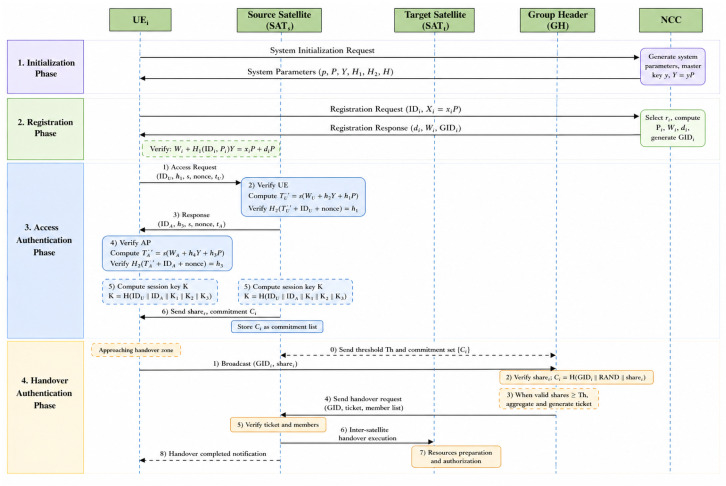
Authentication Flow Chart.

In this section, we design the protocol based on the system model and propose a two-stage access authentication scheme comprising an initialization stage, a registration stage, and an access authentication stage. The complete authentication flow is illustrated in Figure 2.

### 3.1. Initialization Phase

Before communication begins, the NCC should set security parameters. Produces prime numbers *p*, *q*, and q|p−1. The pseudorandom elliptic curve $E(F_{p})$ is generated on $F_{p}$ according to the elliptic curve random generation algorithm, and the basis point is determined according to the arbitrary-order base point discovery algorithm.

Select a safe hash function: $H_{1}:{\left\{0,1\right\}}^{*}\times G\to Z_{q}^{*},H_{2}:{\left\{0,1\right\}}^{*}\to Z_{q}^{*},H:{\left\{0,1\right\}}^{*}\times G^{3}\to {\left\{0,1\right\}}^{k}$. The construction method of $H_{1}$ is to first do the point multiplication operation on the elliptic curve to obtain the point *X*, add the two coordinate values of *X*, modulo *q*, and complete the hash operation. The structure of $H_{2}$ can be directly modulated. The hash function *H* is constructed based on the SHA-256 algorithm.

NCC randomly selects the master private key $y\in Z_{q}^{*}$ of the system, and calculates the master public key Y=yP. The system then exposes the parameters $\left(p,P,Y,H_{1},H_{2},H\right)$, confidential storage *y*.

### 3.2. Registration Phase

The private keys of the UE and AP include partial private keys, long-term private keys, and temporary private keys. Some private keys and declarative public keys are provided by NCC, while the long-term private keys and temporary private keys are generated by users.

The user $ID_{i}$ randomly selects $x_{i}\in Z_{q}^{*}$ as its long-term private key, and generates the corresponding long-term private key $X_{i}=x_{i}P$. The user then sends $X_{i}$ and $ID_{i}$ to the NCC for registration.

For User $ID_{i}$, NCC selects $r_{i}\in Z_{q}^{*}$, and computes $P_{i}=r_{i}P$, $W_{i}=X_{i}+P_{i}$, $d_{i}=r_{i}+yH_{1}\left(ID_{i},P_{i}\right)$. Meanwhile, the NCC assigns a group identity $GID_{i}$ to the user based on geographic location and access characteristics, and transmits $GID_{i}$ together with $d_{i}$ and $W_{i}$ to the user. $W_{i}$ acts as the user’s public key.

After receiving $d_{i}$ and $GID_{i}$, the user computes their complete private key $s_{i}=\left(x_{i},d_{i},a\right)$. User $ID_{i}$ can determine whether the part of the private key assigned to him by NCC is valid by calculating whether equation $W_{i}+H_{1}\left(ID_{i},P_{i}\right)Y=x_{i}P+d_{i}P$ is true.

### 3.3. Access Authentication Phase

The UE and the AP, respectively, prepare the necessary parameters for key agreement by generating them locally and obtaining them from the NCC. In this process, the UE, as the initiator, first calculates a signature using a portion of its long-term private key and a temporary private key, and then sends the signature to the AP via a public channel. Upon receiving it, the AP verifies the signature and sends its own signature information. UE also performs verification to ensure its authenticity. After two rounds of message exchange, the UE and AP complete mutual authentication and key agreement. The elaborate process is listed as:

Step 1: The UE sends an access request to the AP.

In this step, the UE first obtains the elliptic curve parameters *P* and the hash function $H_{2}$. Then, it performs the following computations:

(1) $$T_{U}=aP$$

(2) $$PID_{U}=ID_{U}⊕H_{2}\left(aY\right)$$

(3) $$h_{1}=H_{2}\left(T_{U}+PID_{U}+nonce\right)$$

(4) $$s=a\times {\left(x_{U}+d_{U}+h_{1}\right)}^{-1}$$

Finally, the UE sends the message $\left(PID_{U},h_{1},s,nonce,t_{U},T_{U}\right)$ to the AP to initiate the authentication process.

Step 2: AP verifies the sender’s identity.

Upon receiving the message from the UE, the AP first verifies the freshness of the timestamp $t_{U}$. If the timestamp is outdated or indicates potential replay, the authentication process is immediately terminated to prevent replay attacks. If the timestamp is valid, the AP proceeds with the following computations:

(5) $$ID_{U}=PID_{U}⊕H_{2}\left(yT_{U}\right)$$

(6) $$h_{2}=H_{1}\left(ID_{U}+P_{U}\right)$$

(7) $$T_{U}^{\prime }=s\left(W_{U}+h_{2}Y+h_{1}P\right)$$

Next, the AP verifies the authenticity of the message by checking whether the following condition holds:

(8) $$H_{2}\left(T_{U}^{\prime }+PID_{U}+nonce\right)=h_{1}$$

If the equation holds, the UE is considered legitimate, and the authentication proceeds. Otherwise, the authentication attempt is rejected due to verification failure.

Step 3: AP sends a confirmation message to the UE.

After verifying the UE’s identity, the AP computes a confirmation message for the UE. The following parameters are generated:

(9) $$T_{A}=bP$$

(10) $$h_{3}=H_{2}\left(T_{A}+ID_{A}+nonce\right)$$

(11) $$s=b\times {\left(x_{A}+d_{A}+h_{3}\right)}^{-1}$$

The AP then constructs the response message $\left(ID_{A},h_{3},s,nonce,t_{A}\right)$. This message is sent to the UE to complete the mutual authentication process.

Step 4: The UE verifies the legitimacy of the AP.

Upon receiving the response message from the AP, the UE first verifies the freshness of the timestamp $t_{A}$. If the timestamp is deemed stale, the authentication process is terminated immediately to mitigate replay attacks. If the timestamp is valid, the UE proceeds with the following computations:

(12) $$h_{4}=H_{1}\left(ID_{A}+P_{A}\right)$$

(13) $$T_{A}^{\prime }=s\left(W_{A}+h_{2}Y+h_{3}P\right)$$

The UE then verifies the authenticity of the AP by checking whether the following condition holds:

(14) $$H_{2}\left(T_{A}^{\prime }+ID_{A}+nonce\right)=h_{3}$$

If the equation holds, the AP is considered legitimate, and mutual authentication is successfully achieved. Otherwise, the authentication is deemed unsuccessful, and the process is aborted.

Step 5: Both parties compute the shared key.

After mutual authentication is completed, both the UE and the AP proceed to compute the session key. The key agreement process is designed to ensure that both parties independently derive the same shared key using their respective private parameters and exchanged information. The following computations are performed:

(15) $$K_{U1}=\left(x_{U}+d_{U}+a\right)X_{A}=K_{A1}=x_{A}\left(X_{U}+P_{U}+T_{U}\right)=K_{1}$$

(16) $$K_{U2}=\left(x_{U}+d_{U}+a\right)P_{A}=K_{A2}=d_{A}\left(X_{U}+P_{U}+T_{U}\right)=K_{2}$$

(17) $$K_{U3}=\left(x_{U}+d_{U}+a\right)T_{A}=K_{A3}=b\left(X_{U}+P_{U}+T_{U}\right)=K_{3}$$

(18) $$K=H(PID_{U}||ID_{A}||K_{1}||K_{2}||K_{3})$$

Each party derives the same session key *K* by combining its identity and the intermediate secrets $K_{1},K_{2},K_{3}$. This design ensures resistance against known-key attacks and provides forward secrecy.

Upon successful access authentication, the source satellite executes a key preparation step for subsequent secure group handover. The source satellite generates a unique secret share $share_{i}$ for each authenticated $UE_{i}$ and computes the corresponding hash commitment as $C_{i}$, where RAND is a random nonce allocated by the satellite to enhance anti-counterfeiting and anti-replay capability.

(19) $$C_{i}=H(GID_{i}||RAND||share_{i})$$

The source satellite securely delivers $share_{i}$ and $C_{i}$ to $UE_{i}$ and stores $C_{i}$ locally to form a commitment list for group verification during handover. This mechanism lays the foundation for threshold secret sharing-based group handover and effectively resists malicious handover requests forged by compromised group members or aggregators.

### 3.4. Handover Authentication Phase

To address the signaling storm and efficiency bottlenecks caused by simultaneous handover of massive sensor users in LEO satellite networks, we propose a threshold secret sharing-based group handover authentication protocol optimized for LEO satellite scenarios.

First, users located in adjacent geographical regions are clustered into groups according to their spatial proximity, so that all members in the same group tend to perform handover at approximately the same time. A GH is selected from each group to manage user handovers within the group. In addition, the GH only maintains temporary coordination authority within its local geographical region. Once the GH moves beyond the predefined coverage boundary or the group topology changes significantly, the system triggers dynamic GH reselection and group reconstruction procedures to maintain stable and efficient handover coordination.

During the registration phase, the NCC performs user grouping and assigns a unique GID to each user. During the access authentication phase, after successful mutual authentication, the source satellite distributes a unique secret share $share_{i}$ and a corresponding hash commitment to each user, preparing credentials for subsequent group handover.

When a user moves to the handover zone and is ready to switch to the target satellite, it broadcasts only its GID and secret share $share_{i}$ over the public channel without exposing its real identity, thus preserving user privacy and resisting tracking attacks. The GH monitors such broadcast messages and validates the legitimacy of each received share by checking whether it matches the pre-stored hash commitment.

Once the number of valid secret shares received by the GH exceeds a predefined threshold, the GH aggregates these shares to generate a group handover ticket and sends a unified handover request to the source satellite. Before formal execution, the source satellite verifies the validity of the aggregated ticket and the legitimacy of group members. If the verification passes, the source satellite initiates an inter-satellite group handover procedure.

During the whole process, the source satellite keeps monitoring the status of users and groups in real time. When it detects that a large number of users are approaching the edge of the coverage area and are about to hand over, it sends the handover threshold and complete hash commitment set to the GH in advance. After collecting and verifying sufficient valid shares, the GH triggers the group handover.

The proposed authentication framework assumes that the UE remains within a stable satellite beam coverage region during the execution of the authentication procedure. The authentication completion delay must therefore be shorter than the effective beam residence time to avoid interruption caused by rapid satellite movement.

## 4. Performance Evaluation

In this section, we analyze the protocol’s security requirements and evaluate the computational and communication overhead through experimental simulations.

### 4.1. Security Analysis

To demonstrate the robustness and security of the proposed authentication and key agreement scheme, we conduct a formal security analysis under the DY threat model. The key security properties verified in this analysis include user anonymity and untraceability, mutual authentication, known-key security, master key forward secrecy, key control, and resistance to impersonation attacks under key compromise.

1. User anonymity and untraceability: In the proposed protocol, the UE does not transmit its real identity over the public channel. Instead, a dynamic pseudonym identity $PID_{U}=ID_{U}⊕H_{2}\left(aY\right)$ is generated using the ephemeral random number and the system public key. Since recovering aY without knowledge of either the ephemeral secret *a* or the NCC master private key *y* is computationally infeasible under the elliptic curve discrete logarithm problem, external adversaries cannot derive the real identity of the UE from the transmitted pseudonym. Moreover, the ephemeral random number *a* is independently generated in each authentication session. Consequently, the pseudonym identity changes dynamically across different sessions, preventing attackers from correlating multiple authentication requests belonging to the same UE. Therefore, the proposed scheme achieves both user anonymity and untraceability.
2. Mutual authentication: Mutual authentication ensures that both communication parties can confirm each other’s identity during the protocol execution. The AP verifies access message by checking the freshness of $t_{U}$, recomputing $h_{2}$ and $T_{U}^{\prime }$, and validating the equation:
  (20) $$H_{2}\left(T_{U}^{\prime }+ID_{U}+nonce\right)=h_{1}$$
  If this check passes, the AP is assured of the UE’s legitimacy. UE performs the same identity verification after receiving the response message. Because both sides must verify each other using computations based on ephemeral values and identity-dependent hashes, mutual authentication is achieved. An attacker cannot forge valid responses without knowing session-specific secrets, and any tampering will result in hash mismatches.
3. Known-key security: Known-key security ensures that the compromise of a session key does not compromise the security of past or future session keys. In the proposed scheme, the session key *K* is derived from three independent intermediate values $K_{1},K_{2},K_{3}$, each of which is a function of ephemeral secrets (a,b) and long-term private keys $\left(x_{U},d_{U},x_{A},d_{A}\right)$.Since *a* and *b* are freshly generated for each session, even if an attacker obtains a session key *K*, they cannot derive the ephemeral secrets used in previous or future sessions. Therefore, the scheme satisfies known-key security.
4. Forward secrecy of the master key: Forward secrecy guarantees that the compromise of long-term private keys does not affect the confidentiality of previously established session keys. In the proposed scheme, the session key depends on the ephemeral private values *a* and *b*, which are never transmitted and are independently chosen in each session. Even if an adversary later compromises the long-term private keys $\left(x_{U},d_{U},x_{A},d_{A}\right)$, it remains computationally infeasible to compute previous session keys without access to *a* or *b*, thereby achieving forward secrecy.
5. Security of key control: Key control refers to the property that neither communication party can unilaterally determine the session key. In our scheme, the session key *K* is a function of inputs from both the UE and the AP. Since the session key depends on secrets from both sides and is derived through a cryptographic hash function *H* over shared elliptic curve-based operations, neither party can fully determine or predict the session key independently. This property ensures mutual key control, which is essential in cooperative communication scenarios like LEO satellite network.
6. Resistance to impersonation under key compromise: In real-world scenarios, adversaries may compromise the long-term private keys of either the UE or AP. A secure authentication protocol must prevent adversaries from impersonating legitimate entities, even in such cases. In the proposed scheme, although long-term private keys may be compromised, session-specific ephemeral values *a* and *b* are randomly generated for each session and not reused. These values are essential in computing the authentication tokens and session key. Without access to *a* or *b*, an adversary cannot generate valid authentication messages or derive the correct session key. Therefore, the proposed protocol is resistant to impersonation even under key compromise, provided that ephemeral secrets remain secure.

We compared the security requirements in Table 1 with previous studies, which identified deficiencies in security features. The schemes proposed in [9,12] do not provide forward secrecy. The schemes proposed in [11,12] are vulnerable to impersonation attacks and do not support secure key updates.

### 4.2. Simulation Results

The delay in the access authentication process comprises computational and communication delays. For computational delay, we consider only computationally expensive operations, since splicing and XOR operations have negligible time cost. We use $T_{M}$, $T_{A}$, $T_{H}$, and $T_{s}$ to represent point multiplication, point addition, hash computation, and symmetric encryption operations. We simulated the computational cost of the related operations by performing 1000 measurements and computing the average. The results are recorded in Table 2.

To evaluate the communication costs of the considered schemes, we adopt the total number of bytes transmitted during the access authentication process—under an equivalent security level—as the primary evaluation metric. According to [15], a 160-bit elliptic curve cryptography (ECC) system provides a security level comparable to that of a 1024-bit RSA system. Based on this equivalence, we assume the following bit-lengths for various elements involved in the authentication process: user identity (64 bits), timestamp (32 bits), random number (160 bits), hash digest (160 bits), symmetric encryption input/output (128 bits), large prime p (1024 bits), and elliptic curve point multiplication result (320 bits).

**Figure 3:**
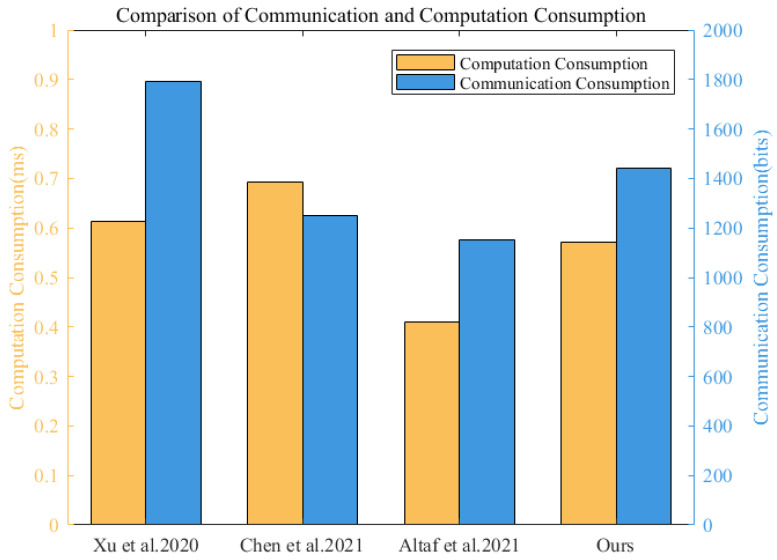
Comparison of Communication and Computation Consumption. Data from Xu et al. 2020, Chen et al. 2021 and Altaf et al. 2021 [10,11,12].

Based on the aforementioned data, we computed the computational and communication overhead of several representative access authentication schemes, as summarized in Table 3. A visual representation of the comparison results is provided in Figure 3.

Our proposed scheme incurs a transmission overhead of 1472 bits in the communication channel and requires approximately 0.572 milliseconds to complete the associated cryptographic computations. Although the scheme presented in [12] exhibits lower computational and communication costs, it suffers from notable security vulnerabilities. In contrast, simulation results indicate that our protocol achieves a favorable balance between security robustness and resource efficiency, demonstrating its suitability for practical deployment in LEO satellite network.

**Figure 4:**
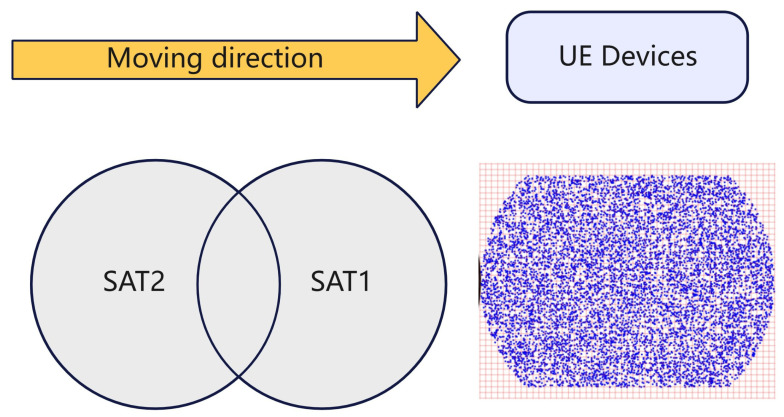
Experimental scenario.

We deploy two satellites to simulate the continuous inter-satellite handover procedure, as illustrated in Figure 4. To better investigate the relationship between the scale of massive sensor users and handover performance, we randomly deploy a large number of UEs within the coverage areas of the two satellites and uniformly assign these UEs to the corresponding satellite service ranges. We assume that all UEs initially access $Sat_{1}$ and then simulate their sequential handover process to $Sat_{2}$. In the standalone handover protocol [16], each UE communicates directly with the target satellite independently to initiate the handover procedure, without any unified coordination or management. To verify the performance difference between the proposed scheme and the standalone handover protocol, we conduct comparative simulation experiments using the standalone handover protocol as the reference. The specific parameters adopted in the simulation experiments are listed in Table 4, covering key indicators such as communication latency, signal transmission rate, and handover success rate, which comprehensively reflect the handover performance of different protocols.

The standalone handover protocol is selected as the primary reference baseline because it represents the conventional independent authentication strategy widely adopted in existing LEO satellite network handover procedures. The objective of this comparison is to evaluate the signaling reduction capability introduced by collaborative grouping rather than to exhaustively benchmark all possible NTN mobility-management solutions.

**Figure 5:**
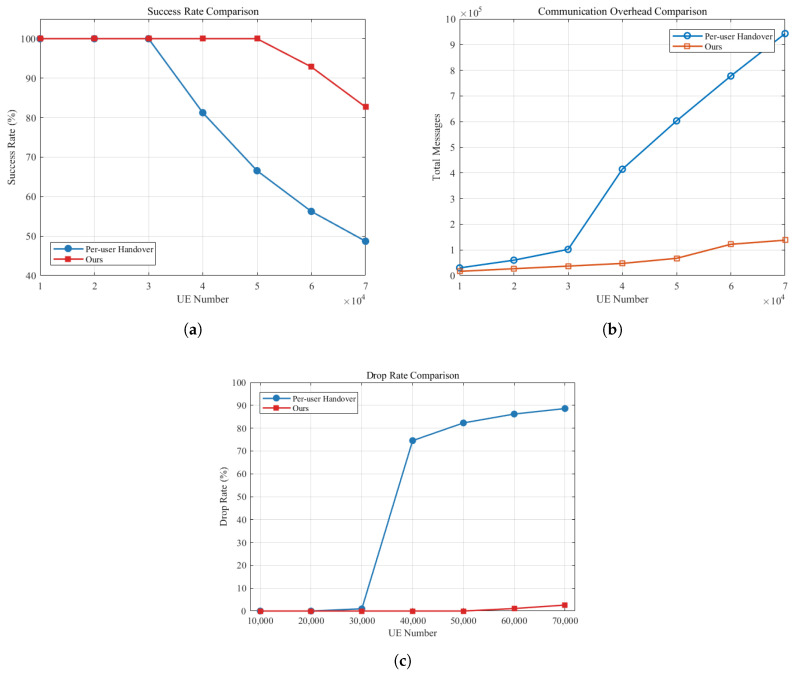
These figures present the comparison of experimental results between the two handover methods. (**a**) Description of the handover success rates of the two handover methods. (**b**) Description of the signaling overhead of the two handover algorithms. (**c**) Description of the drop rate of the two handover algorithms.

The experimental results presented in Figure 5 comprehensively demonstrate the overall performance differences between the two typical handover algorithms under varying user scales. The conventional per-user individual handover protocol can operate stably and handle handover tasks efficiently with a small number of accessing users. When the number of users remains relatively small (10,000–30,000), both the conventional standalone handover protocol and the proposed grouping-based scheme maintain low signaling overhead, stable handover success rates, and extremely low packet drop rates. This is because the signaling load and authentication requests are still within the processing capability of the satellite network, allowing handover procedures to be completed successfully with limited congestion.

However, as the network scale expands sharply, when the number of concurrent access users reaches 40,000, both the total interaction messages and independent user-side signaling messages rise substantially and explosively. The excessive signaling interactions consume massive satellite computing and communication resources, eventually rendering the satellite unable to process massive handover requests in a timely and normal manner. Consequently, the conventional standalone handover protocol experiences a dramatic increase in packet drop rate. In particular, when the user scale reaches 40,000, the drop rate sharply rises to approximately 75%, and continues increasing as the network scale further grows. The overwhelming signaling burden rapidly exhausts satellite processing and communication resources, causing severe congestion, authentication delays, and ultimately large-scale packet losses during the handover process.

In contrast, the proposed grouping-based handover protocol exhibits excellent scalability in large-scale user scenarios. Its total transmission messages and user interaction overhead only increase steadily in an approximately linear trend with the growth of user quantity, while the system consistently maintains a stable and high handover success rate. In the considered simulation setting, the proposed scheme achieves up to 69.81% improvement in handover success rate compared with the standalone handover baseline. Meanwhile, the proposed protocol consistently maintains a very low packet drop rate even under high-density user scenarios.

The underlying reason is that users in adjacent geographical areas are reasonably clustered and managed in groups. This mechanism effectively aggregates distributed individual handover requests, greatly cuts down redundant handover signaling interactions, significantly reduces global signaling overhead, and ultimately alleviates the heavy real-time processing burden of LEO satellite nodes in high-density access scenarios. By transforming massive distributed handover requests into coordinated group-level signaling procedures, the proposed mechanism improves network stability and avoids severe congestion caused by repeated independent authentication interactions.

Therefore, the experimental results indicate that the proposed scheme achieves substantially better robustness, scalability, and transmission reliability than the conventional standalone handover protocol, particularly in dense NTN environments with massive concurrent handovers.

It should be noted that the current simulation results are obtained under a simplified proof-of-concept LEO satellite network scenario consisting of two LEO satellites and geographically correlated user distributions. Therefore, the reported performance improvement mainly reflects the effectiveness of the proposed grouping-based handover mechanism under representative dense-access conditions rather than universal performance gains for all LEO satellite network environments.

### 4.3. Alignment with ITU and NTN Standard Requirements

To further evaluate the practical applicability of the proposed authentication protocol in NTNs, the simulation results are analyzed from the perspective of ITU-recommended performance requirements for future satellite communication systems.

According to ITU-R recommendations for NTN and integrated space–air–ground networks, authentication mechanisms should satisfy several key objectives, including low authentication latency, efficient mobility support, scalable access management, secure handover capability, and low signaling overhead. The experimental results demonstrate that the proposed certificateless authentication protocol fulfills these requirements [17,18]. A detailed compliance evaluation with the major ITU-oriented NTN requirements is summarized in Table 5.

First, the computation cost analysis indicates that the proposed protocol introduces significantly lower computational overhead than pairing-based authentication schemes. Since only elliptic curve scalar multiplication and hash operations are involved, the authentication delay remains sufficiently small for latency-sensitive NTN scenarios. This characteristic is particularly important for LEO satellite systems where users frequently experience beam switching and satellite handovers.

Second, the communication overhead evaluation shows that the proposed protocol requires fewer transmitted bits during both access authentication and handover authentication procedures. Reduced signaling traffic decreases bandwidth consumption and improves spectrum utilization efficiency, which is consistent with the ITU objective of supporting massive machine-type communications in future satellite networks.

Third, the handover simulation results demonstrate that the proposed scheme maintains stable authentication performance under increasing numbers of users. The authentication success rate remains high while the handover delay remains within acceptable limits. These results verify the scalability of the protocol and its suitability for dense satellite-IoT deployment scenarios.

Furthermore, the dynamic pseudonym mechanism effectively protects user identity privacy during authentication and handover procedures. Unlike schemes that directly expose user identities, the proposed protocol periodically updates temporary identifiers, thereby preventing user tracking and enhancing privacy preservation. This property aligns with the security requirements emphasized in emerging NTN security frameworks.

Overall, the simulation results confirm that the proposed scheme achieves a balanced trade-off among security, computational efficiency, communication overhead, and mobility support. Therefore, the protocol is well suited for practical deployment in future LEO satellite communication systems.

### 4.4. System Constraints

To ensure the practical validity of the proposed authentication framework in LEO satellite network environments, the system assumptions, physical-layer constraints, and deployment boundaries are further clarified.

First, the proposed scheme is designed for LEO satellite networks with relatively stable beam coverage during the authentication interval. Specifically, the authentication completion time should remain shorter than the effective beam residence duration of the UE to avoid authentication interruption caused by rapid satellite movement. In addition, the communication channel is assumed to satisfy basic transmission reliability requirements such that authentication signaling can be completed within tolerable propagation delay and packet loss ranges.

Second, the protocol adopts a hierarchical LEO satellite network architecture composed of the NCC, GS, AP, and UE. Secure and trusted communication channels are assumed among NCC, GS, and AP entities, while only the wireless access channel between AP and UE is considered vulnerable under the Dolev–Yao adversarial model.

Third, the proposed scheme assumes that neighboring users can be dynamically organized into collaborative handover groups during large-scale mobility events. Therefore, the grouping mechanism requires sufficient user density and stable local connectivity to guarantee effective group coordination and secret-share aggregation during the handover process.

Furthermore, the proposed protocol mainly focuses on authentication-layer optimization and lightweight signaling management in LEO satellite network systems. Consequently, several lower-layer communication impairments, including severe Doppler shifts, dynamic beam deformation, satellite channel fading, and intermittent inter-satellite link instability, are not explicitly modeled in the current work.

Finally, the current scheme assumes that the registration phase is executed through a secure communication channel and that trusted network entities, including NCC and AP, operate honestly during authentication and handover procedures. More sophisticated threat scenarios involving insider attacks, malicious group heads, or fully decentralized trust management architectures are beyond the scope of this paper.

## 5. Conclusions

This paper proposes an authentication scheme tailored for massive sensor users accessing NTN. To address the challenges of certificate management in NTN scenarios, the scheme adopts a certificateless encryption approach that dispenses with bilinear pairing operations, thereby simplifying the authentication process and reducing computational complexity effectively. In response to the signaling storm caused by the simultaneous handovers of a large number of adjacent sensor users, the scheme introduces a user-group-based handover mechanism that improves handover efficiency and reduces excessive signaling interactions. Additionally, a temporary key mechanism is integrated to ensure the forward secrecy of the master key, further enhancing the security of the authentication process. Experimental results verify that the proposed scheme outperforms existing authentication approaches in both security and performance, fully meeting the application requirements of NTN environments with massive sensor users. It provides a reliable and efficient authentication solution for the stable operation of NTN, laying a solid foundation for the practical application of NTN-related technologies.

## Figures and Tables

**Table 1:** Security comparisons.

Security Features	[9]	[11]	[12]	Ours
Mutual authentication	✓	✓	✓	✓
User anonymity and untraceability	✓	✓	✓	✓
Man-in-the-middle attack	✓	✓	✓	✓
Replay attack	✓	✓	✓	✓
Forward secrecy	✗	✓	✗	✓
Key update	✓	✗	✗	✓
Impersonation attack	✓	✗	✗	✓

**Table 2:** Parameters used.

Notation	User (ms)	NCC (ms)
$T_{m}$	$9.8\times 10^{-2}$	$7.2\times 10^{-2}$
$T_{a}$	$4.5\times 10^{-2}$	$5.5\times 10^{-3}$
$T_{h}$	$1.9\times 10^{-2}$	$1.8\times 10^{-3}$
$T_{s}$	3.58	1.5

**Table 3:** Computation and communication consumption comparisons.

Protocols	UE’s Required Times	NCC’s Required Time	Total Time	Communication Consumption
[10]	$5T_{h}+3T_{m}=0.389$ ms	$5T_{h}+3T_{m}=0.225$ ms	0.614	1792
[11]	$9T_{h}+3T_{m}=0.465$ ms	$6T_{h}+3T_{m}=0.227$ ms	0.692	1248
[12]	$7T_{h}+2T_{m}=0.329$ ms	$5T_{h}+T_{m}=0.081$ ms	0.410	1152
Ours	$3T_{h}+3T_{m}=0.351$ ms	$3T_{h}+3T_{m}=0.221$ ms	0.572	1472

**Table 4:** Simulation parameters.

Parameter	Value
Satellite speed	7.56 km/s
Footprint	25 km
Inter-satellite distance	30 km
Inter-satellite delay	1 ms
Ground–satellite delay	3 ms
Transmission delay	1 μs
Encryption/Decryption	0.1 ms
Signature sign/Verify	0.3 ms

**Table 5:** Quantitative Performance Assessment with Respect to IMT-2030 and NTN Objectives.

ITU-Oriented Requirement	Evaluation Metric	Proposed Result	Compliance
Massive Connectivity	Supported User Scale	40,000+ users	Satisfied
Low-Latency Access	Authentication delay	0.572 ms	Satisfied
Seamless Mobility	Handover success rate	82.70%	Satisfied
Service Continuity	Packet Drop Rate	Low	Satisfied
Scalability	Signaling overhead growth	Approximately linear	Satisfied
Secure Access	Security Analysis	Supported	Satisfied

## Data Availability

The relevant data of this paper can be contacted by lvzhengwei@hust.edu.cn.
